# Causal relationship and mediation effects of immune cells and plasma metabolites in atopic dermatitis: A Mendelian randomization study

**DOI:** 10.1097/MD.0000000000039932

**Published:** 2024-10-11

**Authors:** Kaiwen Yang, Jianqiao Zhong, Dehai Xian

**Affiliations:** a School of Basic Medicine, Southwest Medical University, Luzhou, Sichuan, China.

**Keywords:** atopic dermatitis, causal inference, immune cells, Mendelian randomization, plasma metabolites

## Abstract

Atopic dermatitis (AD) is a chronic inflammatory skin condition with complex etiology involving genetic, environmental, and immunological factors. This study employs Mendelian randomization to explore the causal relationships between immune cell phenotypes and AD, and the mediating effects of plasma metabolites. Using data from European cohorts, we identified 7 immune cell phenotypes significantly associated with AD. Mediation analysis revealed that the alpha-ketobutyrate to 4-methyl-2-oxopentanoate ratio negatively regulates CCR2 on monocytes, while the glycerol to carnitine ratio positively regulates HLA-DR on CD14− CD16− cells. These findings underscore the critical role of metabolic pathways in modulating immune responses and suggest potential dietary and therapeutic interventions for AD management. Further research should consider more diverse populations to validate these findings.

## 1. Introduction

Atopic dermatitis (AD), also known as atopic eczema, is a chronic inflammatory skin condition characterized by dry skin, itching, and recurrent eczematous lesions.^[1,2]^ The prevalence of AD is high worldwide, affecting approximately 20% of children and 10% of adults, making it a significant public health concern.^[3,4]^ The etiology of AD is multifaceted, involving genetic predisposition, environmental factors, and immune system dysregulation.^[5]^

The immune microenvironment in AD is a complex network composed of various immune cells, cytokines, chemokines, and signaling molecules. The activity of Th2 cells is notably increased, producing cytokines such as IL-4, IL-5, and IL-13, which promote IgE production by B cells and recruit and activate eosinophils, leading to allergic reactions and skin inflammation.^[6–8]^ Dendritic cells (DC) also play a crucial role in AD by capturing and processing antigens and modulating immune responses through interactions with T cells.^[3,9]^ Monocytes and macrophages contribute to AD by producing pro-inflammatory cytokines and chemokines, resulting in inflammation and tissue damage. They exacerbate skin inflammation and injury through the release of factors like TNF-α and IL-1β.^[10–13]^ Regulatory T cells are essential for maintaining immune tolerance and controlling excessive inflammatory responses.^[6]^ Dysfunctional Tregs can lead to a loss of control over inflammatory responses, worsening skin inflammation and damage.^[14]^ Reduced Treg suppression results in high levels of inflammatory cytokines, further activating effector T cells, which aggravates skin inflammation and damage.^[15,16]^ Therefore, the immune microenvironment and dysregulation in AD play critical roles in its pathogenesis. Understanding these mechanisms is vital for developing new therapeutic strategies.

Mendelian Randomization (MR) uses genetic variations as instrumental variables (IVs) to mimic randomized controlled trials, thereby reducing confounding factors and reverse causation often present in traditional epidemiological studies. Its advantage lies in inferring causal relationships through genetic variations, avoiding interference from environmental or behavioral factors and revealing more reliable causal links.^[17,18]^ This study aims to explore the specific causal relationships between immune cells and AD onset, as well as the mediating effects of plasma metabolites, using MR. This approach provides scientific evidence for understanding AD mechanisms and developing therapeutic strategies.

## 2. Materials and methods

### 2.1. Study design

**Figure 1.**
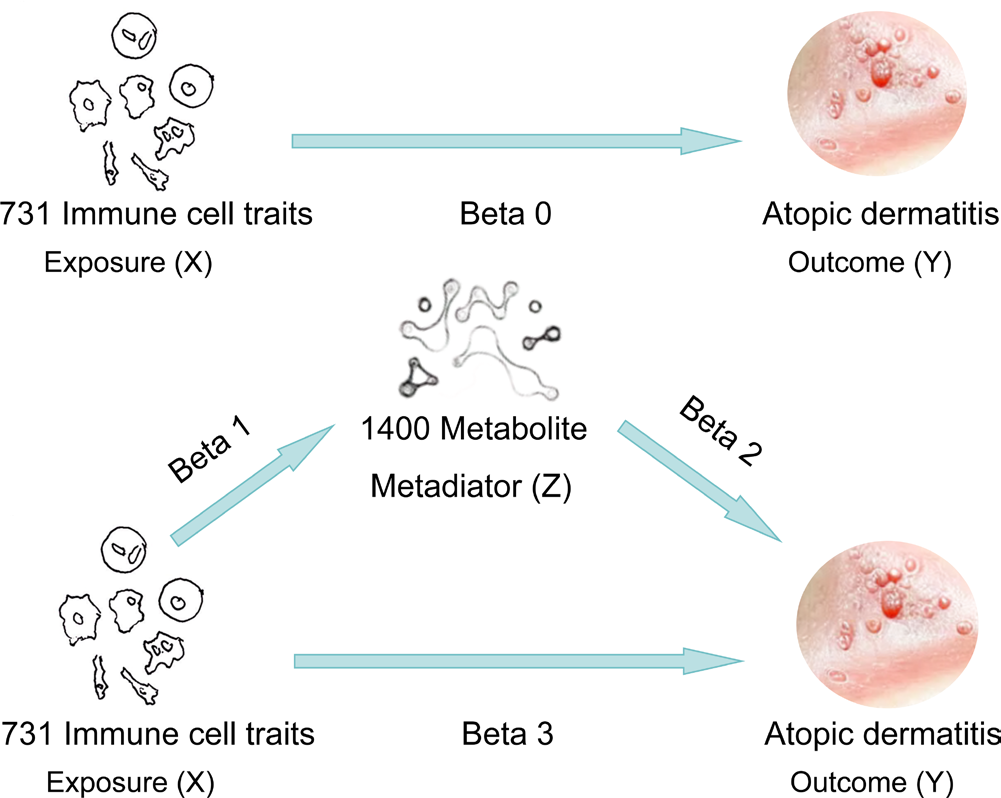
Research framework and hypotheses.

This study utilized bidirectional MR and mediation analysis to investigate the causal relationships between 731 immune cell phenotypes and AD and evaluate the regulatory role of circulating metabolites in this relationship (Fig. 1). First, AD was set as the primary outcome variable, and the 731 immune cell phenotypes were considered potential exposure variables to comprehensively analyze their causal associations. Subsequently, the role of 1400 plasma metabolites as potential mediators in the relationship between AD and immune cell phenotypes was evaluated (Fig. 1). The IVs used in this study needed to meet the following criteria: strongly associated with the exposure factor; not influenced by confounders; independently related to the outcome event. The genome-wide association study (GWAS) data used in the study were sourced from publicly available datasets and approved by respective institutional review boards.

### 2.2. Exposure data sources

#### 2.2.1. 731 Immune cell traits

GWAS data for immune cell traits and AD were obtained from the IEU Open GWAS Project (https://gwas.mrcieu.ac.uk/datasets). For the exposure data, GWAS summary statistics for 731 immune cell traits from a cohort of 3757 individuals of European descent were used.^[19]^ These traits were categorized into 4 groups: 118 absolute cell counts (AC), 192 relative cell counts (RC), 389 median fluorescence intensities (MFI), and 32 morphological parameters (MP). Specifically, the AC, RC, and MFI groups included TBNK (T cells, B cells, natural killer cells), Tregs, T-cell maturation stages, DC, B cells, monocytes, and myeloid cell panels, while the MP group included DC and TBNK panels. These data were derived from a study investigating associations between 731 immune cell phenotypes and 22 million single nucleotide polymorphisms (SNPs) in 3757 individuals of European ancestry (IDs: ebi-a-GCST0001391 to ebi-a-GCST0002121) (Table 1).

**Table 1:** Genome-wide association study data for immune cells, plasma metabolites, and AD.

Exposure or outcome	Sample size	Population	Data source	PMID
Immune cell types	3757	European	https://pubmed.ncbi.nlm.nih.gov/32929287/	32929287
Plasma metabolites	8299	European	https://pubmed.ncbi.nlm.nih.gov/36635386/	36635386
Atopic dermatitis	22,474	European	https://pubmed.ncbi.nlm.nih.gov/34454985/	34454985

### 2.3. 1400 Plasma metabolites

GWAS data for plasma metabolites were obtained from the Canadian longitudinal study on aging, a cohort study involving 8299 participants.^[20]^ This GWAS covered 1091 blood metabolites and 309 metabolite ratios, totaling 1400 unique metabolites (Table 1).

### 2.4. Atopic dermatitis

The GWAS data for AD were sourced from patients in the UK, Finland, and Estonia, including 22,474 cases and 774,187 controls^[21]^ (Table 1).

### 2.5. Selection of instrumental variables

**Figure 2.**
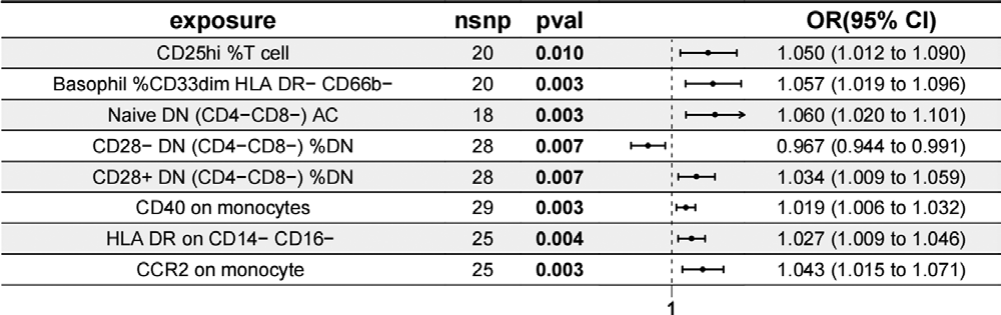
Forest plot illustrating the causal relationship between 8 immune cell types and Atopic dermatitis (AD) using the inverse-variance weighted (IVW) method in Mendelian randomization (MR).

The selection of valid IVs was based on 3 fundamental assumptions (Fig. 2). First, SNPs associated with the exposure were identified with a *P*-value threshold of < 1 × 10^−5^, linkage disequilibrium *r*^2^ < 0.1, and a distance > 1 Mb. Different screening criteria were set for various exposures to ensure a sufficient number of SNPs.^[22]^ Next, to minimize confounding, SNPs related to outcome risk factors were excluded by searching in PhenoScanner V2.^[23]^ The exposure-related SNPs were then extracted from the outcome GWAS data. We employed the Steiger filter to test the causal direction of each SNP between the exposure and outcome, removing SNPs that explained more variance in the outcome than in the exposure.^[24]^ Finally, the MR-PRESSO method was applied to identify and remove potential outlier SNPs. The strength of the IVs was assessed by calculating the *R*^2^ and *F*-statistics, with an *F*-statistic > 10 indicating a low likelihood of weak instrument bias.^[25]^

### 2.6. Mendelian randomization analysis

Bidirectional 2-sample MR analyses were conducted to elucidate the relationships between immune cell traits and AD and explore the potential mediating effects of 1400 metabolites on these traits. The core method used was the inverse-variance weighted (IVW) approach, recognized for its robustness in MR studies.^[26]^ To enhance the reliability of our findings, supplementary analyses were performed using the weighted median and MR-Egger regression techniques. The intercept from MR-Egger regression was examined to check for directional pleiotropy. Additionally, the MR-PRESSO method was used to detect outliers.^[27,28]^ Cochran *Q* test was applied to assess heterogeneity among datasets, and in the presence of significant heterogeneity, random-effects IVW was adopted as the primary analysis method. Primary MR results were considered significant at *P* < .05.

## 3. Results

### 3.1. Overall causal effect of circulating immune cells on atopic dermatitis

In our MR analysis, we identified 8 immune cell traits significantly associated with AD (Fig. 2). These include 1 cDC trait, 4 T-cell maturation traits, 2 monocyte traits, and 1 myeloid cell trait (Fig. 2). Notably, the *P*-value for CCR2 on monocyte was 0.003 with an OR of 1.043 (1.015–1.071), and the *P*-value for HLA-DR on CD14− CD16− was 0.004 with an OR of 1.027 (1.009–1.046), suggesting a potential increased risk for AD.

We further evaluated heterogeneity using IVW and MR-Egger methods. The results indicated that the intercepts for both MR-Egger and IVW for the 8 immune cell phenotypes were not statistically significant, suggesting that the findings were not influenced by any potential bias due to heterogeneity (Table 2). Additionally, horizontal pleiotropy was assessed using MR-Egger, revealing no significant results (*P* > .05), indicating no genetic pleiotropy bias. Sensitivity analysis through leave-one-out method confirmed the robustness of our findings, with no SNP showing an undue influence on the overall causal relationship (Table 2).

**Table 2:** Mendelian randomization analysis of immune cell subsets and monocyte markers in the association with AD: Cochran *Q* test and MR-Egger results.

Exposure	Outcome	Cochran *Q* test		MR-Egger	
		MR-Egger (*P*-value)	IVW (*P*-value)	Intercept	*P*-value
CD25hi %T cell	Atopic dermatitis	.016	.020	−0.004	.653
Basophil %CD33dim HLA-DR- CD66b-	Atopic dermatitis	.001	.002	0.009	.389
Naive DN (CD4− CD8−) AC	Atopic dermatitis	.234	.223	0.008	.307
CD28− DN (CD4− CD8−) %DN	Atopic dermatitis	.260	.283	−0.003	.501
CD28+ DN (CD4− CD8−) %DN	Atopic dermatitis	.260	.283	0.003	.524
CD40 on monocytes	Atopic dermatitis	.166	.195	−0.002	.731
HLA-DR on CD14− CD16−	Atopic dermatitis	.136	.163	0.002	.695
CCR2 on monocyte	Atopic dermatitis	.193	.220	0.005	.581

Reverse MR analysis was performed on the 8 immune cell phenotypes associated with AD, ruling out phenotypes with reverse causal effects. The analysis indicated no reverse causality for these 8 immune cell traits (*P* > .05) (Supplementary Table 1, http://links.lww.com/MD/N698). Further analysis of immune cell phenotypes related to AD screened those with consistent directional effects (OR > 1 or OR < 1) across 5 different MR analysis methods. CD28− DN (CD4− CD8−) %DN was excluded, identifying 7 immune cell phenotypes associated with AD for subsequent mediation analysis.

### 3.2. Mediation effect of plasma metabolites on immune cells and atopic dermatitis risk

**Figure 3.**
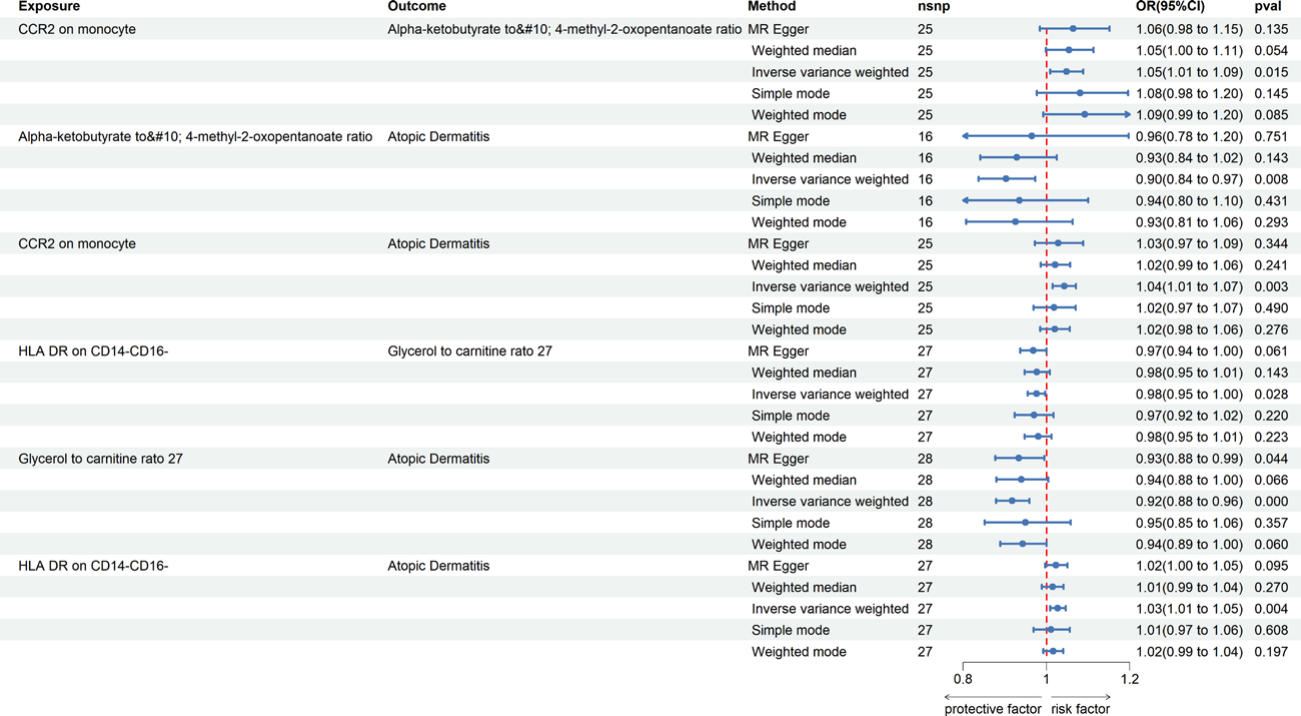
Forest plot depicting the associations between immune cell surface markers (CCR2 on monocytes and HLA-DR on CD14− CD16− monocytes), plasma metabolite ratios (alpha-ketobutyrate to 4-methyl-2-oxopentanoate, and glycerol to carnitine), and AD.

To elucidate the potential mechanisms by which immune cells influence AD, we performed a 2-sample MR analysis. We first analyzed the relationship between immune cells and plasma metabolites (Beta1) and then analyzed the causal relationship between plasma metabolites and AD (Beta2). After calculating their respective mediation effects, we identified 2 pairs of plasma metabolites that mediate the relationship between immune cell phenotypes and AD (Fig. 3).

The analysis revealed that the ratio of Alpha-ketobutyrate to 4-m ethy l-2-oxopentanoate negatively mediates the effect of CCR2 on monocyte on AD (mediation effect, ME = −0.00478; mediation proportion, MP = −11.5%). Additionally, the ratio of glycerol to carnitine positively regulates the effect of HLA-DR on CD14− CD16− on AD (ME = 0.00209; MP = 7.8%) (Table 3).

**Table 3:** Mediation analysis of CCR2 and HLA-DR on monocytes and CD14− CD16−cells in the association with AD via metabolite ratios.

Exposure	Intermediary	Outcome	Mediated effect	Mediated proportion	*P*-value
CCR2 on monocyte	Alpha-ketobutyrate to 4-methyl-2-oxopentanoate ratio	Atopic dermatitis	−0.00478 (−0.00902, −0.000536)	−11.5% (−21.6%, −1.28%)	.027283193
HLA-DR on CD14− CD16−	Glycerol to carnitine ratio	Atopic dermatitis	0.00209 (0.000147, 0.00403)	7.8% (0.548%, 15%)	.035022253

**Figure 4.**
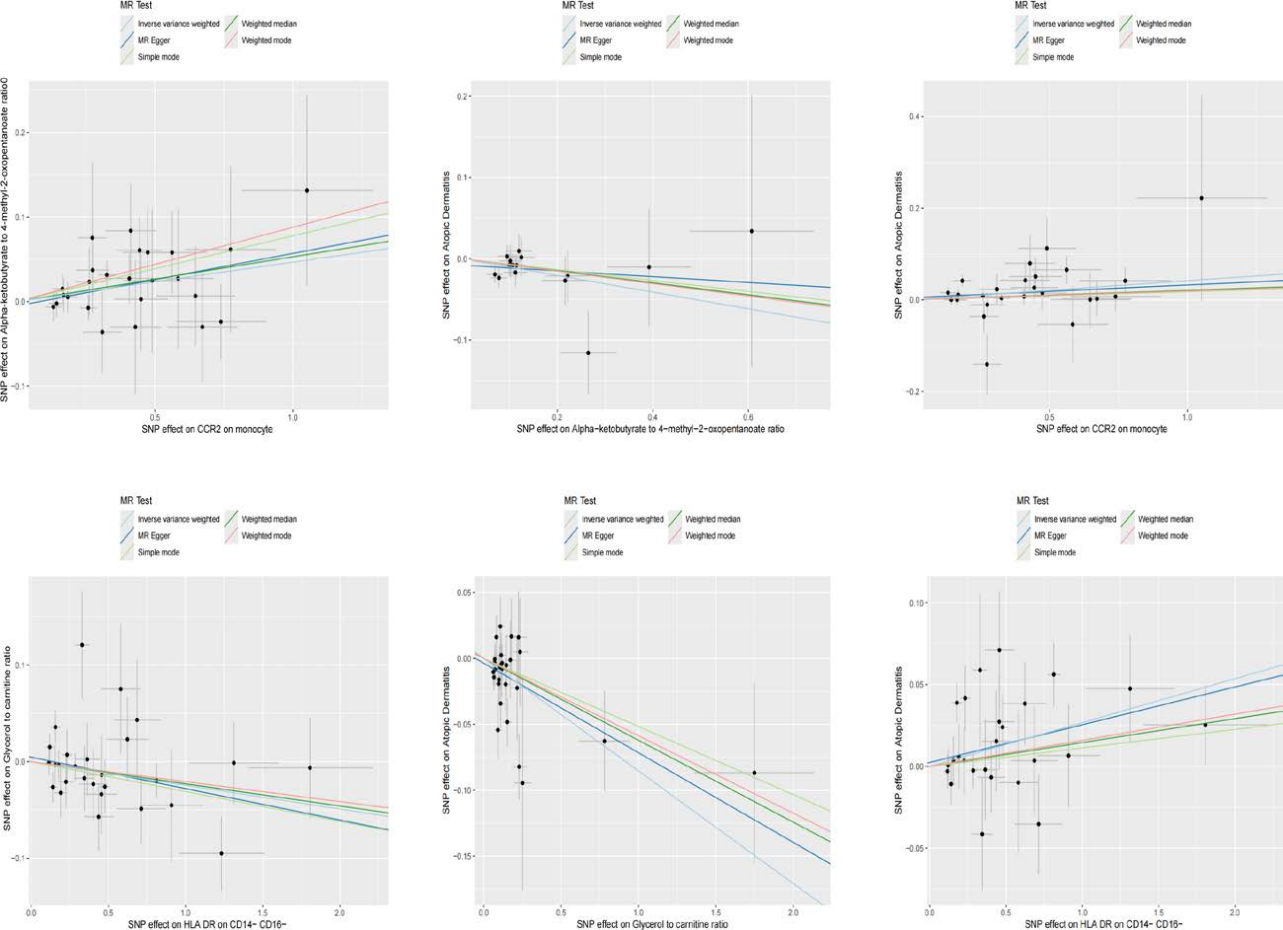
Scatter plot depicting the relationships among immune cells, plasma metabolites, and AD.

**Figure 5.**
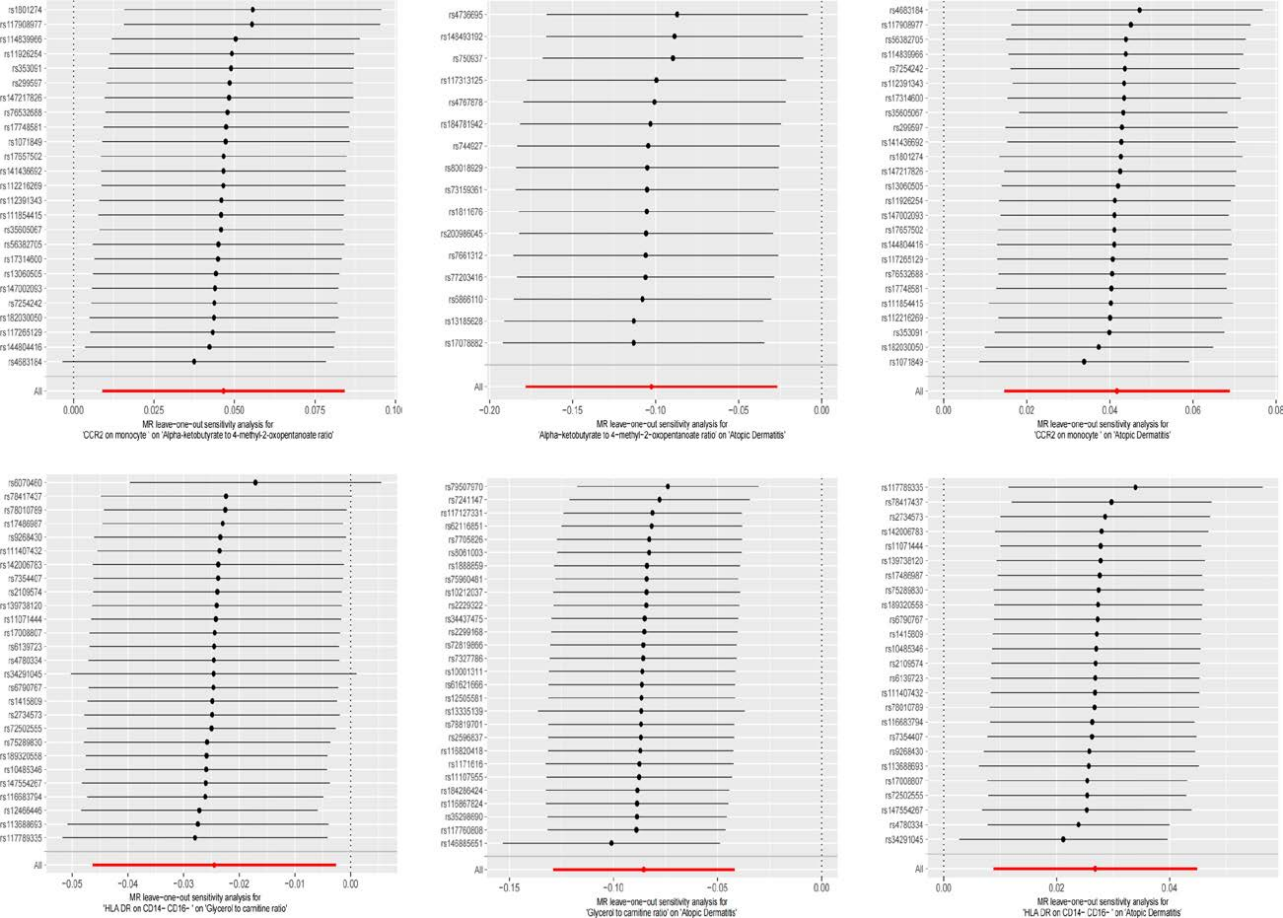
Sensitivity analysis of the associations between immune cells, plasma metabolites, and AD, conducted using the leave-1-out method.

Similar to previous analyses, heterogeneity was assessed using IVW and MR-Egger methods, with results indicating no statistically significant intercepts, thus ruling out any potential bias due to heterogeneity. MR-Egger assessment of horizontal pleiotropy also indicated no genetic pleiotropy bias (Table S2, Supplemental Digital Content, http://links.lww.com/MD/N699). Leave-1-out sensitivity analysis further validated the robustness of the findings, with no SNP showing an undue influence on the overall causal relationship (Figs. 4 and 5).

## 4. Discussion

In our MR study, results indicated a causal relationship between 7 immune cell phenotypes and AD. Further mediation analysis revealed that 2 cell phenotypes have a causal relationship with AD through 2 plasma metabolites, but these metabolites exert opposite regulatory effects. Specifically, the alpha-ketobutyrate to 4-methyl-2-oxopentanoate ratio negatively regulates CCR2 on monocytes in AD, while the glycerol to carnitine ratio positively regulates HLA-DR on CD14− CD16− cells in AD.

CCR2 is a major chemokine receptor on monocytes, responsible for guiding their migration to sites of inflammation. Inflammatory diseases such as AD are closely associated with increased CCR2 expression and exacerbated inflammatory responses. Studies have shown that approximately 90% of peripheral blood monocytes in AD patients express CCR2, and these CCR2-positive cells also express CD163, a marker for monocytes/macrophages.^[29]^ This aligns with our findings. Alpha-ketobutyrate plays a role in energy production and the synthesis of glucose and fatty acids and serves as a precursor for amino acids like isoleucine. Meanwhile, 4-methyl-2-oxopentanoate is crucial in energy generation and the synthesis of cholesterol and other isoprenoids.^[30,31]^ Our study suggests that the alpha-ketobutyrate to 4-methyl-2-oxopentanoate ratio represents energy metabolism as well as the metabolism of alanine, serine, and isoleucine, thereby regulating CCR2 expression and activity, affecting monocyte migration and inflammatory responses.^[32–35]^

High HLA-DR expression enhances the function of antigen-presenting cells (APCs), promoting T-cell activation and differentiation. AD is a T-cell-mediated inflammatory disease, where increased HLA-DR expression may lead to excessive T-cell activation, sustaining and aggravating the inflammatory response.^[35–39]^ Moreover, high HLA-DR expression releases various inflammatory mediators such as IL-1, IL-6, and TNF-α, activating Th2-type immune responses, which leads to recurring and spreading inflammation.^[40,41]^ Although there is no direct evidence linking fatty acid metabolism with HLA-DR expression, our study indicates that the glycerol to carnitine ratio not only reflects the energy metabolism status but also indirectly influences the production of fatty acid metabolites (e.g., prostaglandins and leukotrienes), thus regulating immune cell function and HLA-DR expression.^[42,43]^

Our findings highlight that these metabolites primarily impact the causal effect of monocytes in AD. The functions and behaviors of CD14− CD16− HLA-DR + cells and CCR2 + monocytes are indeed modulated by energy metabolism status. Metabolic pathways such as glycolysis and fatty acid oxidation, along with key regulatory factors like AMPK and mTOR, play vital roles in regulating these immune cells’ functions. These metabolic mechanisms influence not only cellular energy supply but also gene expression and functional characteristics, affecting immune responses and inflammatory processes. Moreover, specific metabolites can be regulated to enhance or diminish their mediating effects, thereby modulating the activity of inflammatory cells.^[43–45]^

These insights propose potential treatment strategies for AD, including dietary and metabolic interventions to reduce inflammatory mediator production and enhance antigen presentation capability. For instance, reducing the intake of sulfur-containing amino acids can decrease alpha-ketobutyrate production, while increasing the intake of branched-chain amino acids (e.g., isoleucine) can boost 4-methyl-2-oxopentanoate production.^[46,47]^ Additionally, drug interventions and gene editing technologies, such as CRISPR-Cas9, can regulate the expression of key enzymes in relevant metabolic pathways, offering effective AD treatments.^[32,35,48–50]^

While our study offers new insights, it also faces certain challenges, primarily due to the data being based on a European population. Future research should incorporate more diverse ethnic samples and increase sample sizes to enhance the persuasiveness of our findings.

## Acknowledgments

We thank the participants and researchers involved in this study. We acknowledge the UK Biobank GWAS Catalog, and Open GWAS for providing GWAS summary statistics.

## Author contributions

**Conceptualization:** Kaiwen Yang, Jianqiao Zhong, Dehai Xian.

**Data curation:** Kaiwen Yang, Jianqiao Zhong, Dehai Xian.

**Formal analysis:** Kaiwen Yang, Jianqiao Zhong, Dehai Xian.

**Funding acquisition:** Kaiwen Yang, Jianqiao Zhong.

**Investigation:** Kaiwen Yang, Jianqiao Zhong.

**Methodology:** Kaiwen Yang.

**Project administration:** Kaiwen Yang.

## Supplementary Material


